# State of microbeads in facial scrubs: persistence and the need for broader regulation

**DOI:** 10.1007/s11356-025-36341-3

**Published:** 2025-04-07

**Authors:** Christelle Not, Kayi Chan, Mandy Wing Kwan So, Woody Lau, Lilia Tsz-Wing Tang, Coco Ka Hei Cheung

**Affiliations:** 1 Department of Earth Sciences, University of Hong Kong, Pokfulam, Hong Kong, SAR China; 2 Swire Institute of Marine Science, University of Hong Kong, Cape d’Aguilar, Hong Kong, SAR China; 3 School of Life Sciences, The Chinese University of Hong Kong, Hong Kong, SAR China

**Keywords:** Microplastic, Microbeads, Ban, Spectroscopy, Global plastic pollution, PCCPs

## Abstract

Microbeads are small spherical plastic particles used as exfoliants in personal care products. Unfortunately, they have been found in the marine environment and are considered a significant contributor to global plastic pollution. In response, several countries have implemented microbead bans over the last few years. Here, we examined the exfoliant (scrubbing particles) composition of 28 facial scrubs from different regions in the presence and absence of microbead bans. We identified that over half of the exfoliant types identified in this study are microbeads, revealing the persistence of microbeads across various stages of microbead ban implementation. In regions with full bans, six out of eight products still contain microbeads, with some containing up to 6298 ± 1543 beads per gram of facial wash, suggesting the need for stronger legislation enforcement. We also identified challenges in distinguishing between microbeads composed of conventional plastics and synthetic waxes by Fourier-transform infrared spectroscopy. This study highlights the need to broaden the scope of microbead bans to include synthetic waxes, as they are not currently regulated. These findings underscore the importance of a broader and clearly articulated definition of microbeads in legislation to guide industry formulation and consumer choice of microbead-free products.

**Supplementary Information:**

The online version contains supplementary material available at 10.1007/s11356-025-36341-3.

## Introduction

Larger plastic debris (> 5 mm), known as macroplastics, fragment into microplastics (≤ 5 mm) under the influence of UV radiation, wind, and microbial activity (Andrady 1994, 2011; Corcoran et al. 2009; Efimova et al. 2018; Min et al. 2020; So et al. 2022; Cheung and Not 2024a, 2024b). These microplastics, identified as secondary microplastics, represent a majority of the microplastics found in the ocean. Meanwhile, 15–31% of microplastics originate from primary sources (primary microplastics), where plastic pieces are intentionally manufactured to be smaller than 5 mm in size (Boucher and Friot 2017). Given their ubiquitous presence and minute size, microplastics are readily available to be ingested by a wide range of marine organisms from various trophic levels with different foraging strategies (Ritchie and Roser 2018; So et al. 2023).

Microbeads, spherical beads used as scrubbing agent in personal care or cosmetic products (PCCPs), are one of the most commonly found primary microplastic in the oceans (Eriksen et al. 2013; Isobe et al. 2015; Cheung and Fok 2016). Since the late 1990s, these small plastic exfoliants, typically spherical in shape with a diameter around 200–400 μm and mainly composed of polyethylene (PE) or polypropylene (PP), have been replacing natural materials such as pumice, oatmeal, and walnut husk in exfoliating skin cleansers (Fendall and Sewell 2009; New York State Attorney General 2014; Dauvergne 2018). Napper et al. (2015) estimated that up to 94,500 microbeads could be washed down the drain in a single use, with 2–10% escaping wastewater treatment, resulting in a non-negligible release into the marine environment (Murphy et al. 2016; Duis and Coors 2016). Conversely, sludge cakes that capture microbeads during the waste treatment process are utilized as agricultural fertilizers, allowing microbeads to enter the marine environment via runoff (Rochman et al. 2015; Boucher et al. 2016; Murphy et al. 2016; So et al. 2018). Increasing public awareness and concern about the unnecessary nature of these single-use plastic microbeads has prompted action from manufacturers and policy-makers. Multinational companies including Johnson and Johnson, Procter and Gamble, Target Corporation, The Body Shop, and L’Oréal have pledged to phase out microbeads from their products (Rochman et al. 2015). Full bans on the manufacture, import, and sale of products containing microbeads took effect in the United States (US), Canada, New Zealand, and the United Kingdom (UK) in 2018 and 2019. European countries including Finland, France, Iceland, Ireland, Italy, Luxemburg, Norway, and Sweden followed with full bans in 2020. Dauvergne (2018) estimates that the combined effect of these bans will lead to a 1–2% reduction in microbead emission into the environment over the next decade.

In this study, we acquired a total of 28 facial scrub products sourced from various regions that are at different stages of implementing bans on microbeads. Our primary objectives were to (1) investigate the prevalence of microbeads in the market, (2) evaluate the effect of legislations regulating microbeads in PCCPs, and (3) identify potential gaps in the legislation to advance efforts to combat microbead pollution. More precisely, we first aim to assess how widely microbeads are still present in these products, despite the growing awareness and regulatory efforts aimed at reducing their use. Secondly, we analyzed the impact of existing laws on the formulation and sale of facial scrubs, determining whether these regulations have successfully reduced the presence of microbeads in the marketplace. Finally, by examining the current regulatory frameworks, we seek to uncover shortcomings or inconsistencies that may hinder effective combat against microbead pollution. This insight will help inform future legislative actions aimed at protecting the environment and public health. To our knowledge, such evaluation has not been done for personal care products bought in different parts of the world.

## Materials and methods

### Facial scrub samples

We purchased 28 facial scrubs representing eight brands and seven regions including Canada, Germany, Hong Kong SAR, Italy, Japan, Scotland (UK), and the United States (US). Of the 28 scrubs, eight bottles were purchased in a region where microbead ban legislation was implemented (Canada, US and UK; CA4-CA6, US5, UK1-UK4), nine bottles were purchased in a region where ban legislation was announced but yet to be implemented (Canada, US and Italy; CA1-CA3, US1-US4, IT1, IT2), and 11 bottles were purchased in a region with no legislation in place (Hong Kong SAR, Japan and Germany; HK1-HK4, JP1-JP4, DE1-DE3) (Table 1) at the time of the purchases.

**Table 1:** List of the 28 facial scrub samples tested in this study, with details of their region of purchase, product brand, date of purchase, ban implementation stage (at the time of purchase), microbead detection, and microbead abundance

Sample	Region of purchase	Brand	Date of purchase	Ban implementation stage (at purchase)	Microbeads detected	Microbead abundance (per g of product)
HK1	Hong Kong SAR	Brand 6	12/2018	No ban	Yes	12,284 ± 1300
HK2	Hong Kong SAR	Brand 2	12/2018	No ban	-	-
HK3	Hong Kong SAR	Brand 1	12/2018	No ban	-	-
HK4	Hong Kong SAR	Brand 2	12/2018	No ban	Yes	86 ± 10
JP1	Japan	Brand 7	01/2019	No ban (Voluntary)	Yes	4062 ± 1777
JP2	Japan	Brand 1	01/2019	No ban (Voluntary)	-	-
JP3	Japan	Brand 6	06/2019	No ban (Voluntary)	-	-
JP4	Japan	Brand 8	06/2019	No ban (Voluntary)	Yes	839 ± 73
DE1	Germany	Brand 2	01/2019	No ban (Voluntary)	Yes	64 ± 12
DE2	Germany	Brand 5	01/2019	No ban (Voluntary)	-	-
DE3	Germany	Brand 3	01/2019	No ban (Voluntary)	-	-
CA1^a^	Canada	Brand 1	12/2018	Announced	Yes	6188 ± 404
CA2^b^	Canada	Brand 2	12/2018	Announced	Yes	89 ± 3
CA3	Canada	Brand 3	12/2018	Announced	-	-
US1	United States	Brand 4	01/2019	Announced	Yes	133 ± 19
US2	United States	Brand 1	01/2019	Announced	Yes	2891 ± 762
US3^c^	United States	Brand 2	01/2019	Announced	Yes	26 ± 1
US4	United States	Brand 5	01/2019	Announced	-	-
IT1	Italy	Brand 3	12/2018	Announced	-	-
IT2	Italy	Brand 5	12/2018	Announced	-	-
CA4^b^	Canada	Brand 2	01/2020	Implemented	Yes	81 ± 10
CA5	Canada	Brand 4	01/2020	Implemented	Yes	124 ± 5
CA6^a^	Canada	Brand 1	01/2020	Implemented	Yes	6298 ± 1543
US5^c^	United States	Brand 2	01/2020	Implemented	Yes	24 ± 5
UK1	Scotland	Brand 2	01/2019	Implemented	Yes	5671 ± 1970
UK2	Scotland	Brand 1	01/2019	Implemented	Yes	7851 ± 1328
UK3	Scotland	Brand 5	01/2019	Implemented	-	-
UK4	Scotland	Brand 3	01/2019	Implemented	-	-

^a, b, c^: Paired products, which were the same products from the same brand purchased in the same region before and after microbead bans were enacted

### Sample preparation

We dissolved 1–2 g of facial scrub in boiling water and extracted insoluble particles using gravity filtration on an 11-μm filter paper (Cheung and Fok 2017). We dried samples to a constant weight at 55℃ and gently scraped off all insoluble particles from the filter paper into a glass petri dish to allow for sorting by colour and shape as well as quantification. Three replicates of the above procedure were performed for each bottle, resulting in a total of 84 samples.

### Sample analysis and quantification

Based on shape and colour, representative samples for each type of insoluble particle were selected in triplicate. Particle composition was identified using the Bruker LUMOS II microscope in attenuated total reflectance mode (FTIR-ATR). Each particle was measured with 32 scans per measurement, generating a mean spectrum in the range of 600–4000 cm^−1^ with a resolution of 4 cm^−1^ (Cheung and Fok 2016). Using the Bruker OPUS software, sample spectra were compared to reference spectra contained in the ATR FTIR Complete Vol. 1–4 and ACLAB.S01 libraries. Polymer identification was based on the highest hit quality index (HQI) returned. Each type of particle identified as plastic by FTIR-ATR was also measured by Raman spectroscopy using the point acquisition mode of a Renishaw inVia confocal Raman microscope (Wotton-under Edge, UK) with a Leica 10 × objective lens and a 785-nm edge laser. Raman spectra were obtained using 0.05–0.5% laser power for 10 s in a Raman shift range of 603–1717 cm^−1^. Standard baseline correction, cosmic ray removal, and smoothing was performed using the Renishaw WiRE 5.5 software. Sample Raman spectra were compared to reference spectra of the Renishaw Polymeric Materials Database. Polymer identification was based on a matching index value > 0.7 (Leung et al. 2021).

Particles identified as synthetic polymers by FTIR and Raman analyses were classified as microbeads, which were then counted using the multipoint tool in the Image J software on scanned images of the petri dishes. For each type of microbead, 30 microbeads were selected randomly and the diameter was measured using a Zeiss Stemi 305 Stereo Zoom microscope and Zeiss Labscope software. For non-spherical microbeads, the maximum span was measured instead.

## Results

Insoluble particles (i.e. exfoliants) in each facial scrub sample were characterized into types based on their shape, colour, and composition. In total, 42 types of exfoliants were identified from 28 bottles of facial scrub. Of these 42 types, 20 were identified by FTIR as non-plastic polymers (including cotton, cotton/flax, cellulose swab, castor oil hydrogenated, and agarose). Most of the non-plastic exfoliants were spherical in shape (17 out of 20 types, 85%). Out of the 20 types of non-plastic exfoliants, nine were white (45%), eight were blue (40%), two were orange (10%), and one was black (5%) in colour (Table 2).

**Table 2:** Characterization of exfoliants extracted from 28 facial scrub samples, including the presence of microbeads, their shape, colour, chemical composition, and average size

Sample	No. of types of exfoliants	No. of types of microbeads	Total no. of microbeads (per g of product)	Exfoliant characteristics					
				Microbead	Shape	Colour	Polymer identification by FTIR	Polymer verification by Raman	Average microbead size (mm)
CA1^a^	2	2	6188 ± 404	✓	Round	White	Polyethylene wax	PE	0.194 ± 0.065
				✓	Round	Orange	PE	PE	0.413 ± 0.107
CA2^b^	1	1	89 ± 3	✓	Round	Blue	PE	PE	0.427 ± 0.133
CA3	2	0	/		Round	Blue	Cotton + flax (60:40)	/	/
					Round	White	Cotton	/	/
CA4^b^	1	1	81 ± 10	✓	Round	Blue	PE	PE	0.432 ± 0.124
CA5	2	1	124 ± 5	✓	Round	Purple	PE	PE	0.352 ± 0.077
					Round	White	Cotton flax	/	/
CA6^a^	2	2	6298 ± 1543	✓	Round	Orange	PE	PE	0.466 ± 0.111
				✓	Round	White	Dyneema/PE	PE	0.305 ± 0.109
US1	2	1	133 ± 19	✓	Round	Purple	Microcrystalline wax	PE	0.364 ± 0.085
					Round	White	Cellulose swab	/	
US2	2	2	2891 ± 762	✓	Round	White	PE	PE	0.309 ± 0.053
				✓	Round	Blue	PE	PE	0.361 ± 0.092
US3^c^	2	1	26 ± 1	✓	Round	Blue	PE	PE	0.724 ± 0.114
					Round	White	Cellulose swab	/	/
US4	1	0	/		Round	Blue	Castor oil, hydrogenated	/	/
US5^c^	2	1	24 ± 5	✓	Round	Blue	PE	PE	0.648 ± 0.08
					Round	White	Cellulose swab	/	/
HK1	2	2	12403 ± 1301	✓	Round	White	Tetracontane	PE	0.555 ± 0.131
				✓	Irregular	White	Tetracontane	PE	0.593 ± 0.130
HK2	1	0	/		Irregular	Orange	Agarose	/	/
HK3	2	0	/		Irregular	Black	Cotton + flax (60:40)	/	/
					Irregular	White	Cotton	/	/
HK4	1	1	86 ± 10	✓	Round	Blue	PE	PE	0.436 ± 0.113
JP1	1	1	4062 ± 1777	✓	Irregular	White	PE	PE	0.525 ± 0.152
JP2	1	0	/		Round	Orange	PFR Rayon, Rayon fiber	/	/
JP3	1	0	/		Round	White	1,2,3-Propantyltridocosanoate	/	/
JP4	1	1	839 ± 73	✓	Round	White	Vexar plastic netting, Olefin fiber	PE	0.590 ± 0.125
IT1	2	0	/		Round	Blue	Cotton + flax (60:40)	/	/
					Round	White	Cotton + flax (60:40)	/	/
IT2	1	0	/		Round	Blue	Castor oil, hydrogenated	/	/
DE1	1	1	64 ± 12	✓	Round	Orange	PE	PE	0.499 ± 0.16
DE2	1	0	/		Round	Blue	Mobil SHC (synthetic for high temperature, roiling oil)	/	/
DE3	1	0	/		Round	Blue	Cotton	/	/
UK1	2	2	5671 ± 1970	✓	Round	Green	PE	PE	0.500 ± 0.083
				✓	Round	White	Copolymer EPDM	Polyvinyl stearate/PE	0.229 ± 0.027
UK2	2	2	7851 ± 1328	✓	Round	White	PE	PE	0.237 ± 0.065
				✓	Round	Orange	PE	PE	0.438 ± 0.118
UK3	1	0	/		Round	Blue	Pantone black natural 10009 (dried)	/	/
UK4	2	0	/		Round	Blue	Cotton + flax (60:40)	/	/
					Round	White	Cellulose swab	/	/

^a, b, c^: Paired products, which were the same products from the same brand purchased in the same region before and after microbead bans were enacted

The remaining 22 types of insoluble particles were identified by FTIR as plastics (PE, olefin, and copolymer EPDM) or plastic-like polymers (PE wax, microcrystalline wax, and tetracontane) (Hartmann et al. 2019). Raman analysis also confirmed that the 22 types of insoluble particles were indeed plastics (PE and Polyvinyl stearate/PE). The majority of these microbeads were spherical in shape (20 out of 22 types, 91%) and came in a wide range of colours of which nine were white (41%), six were blue (27%), four were orange (18%), two were purple (9%), and one was green (5%) (Table 2).

**Fig. 1:**
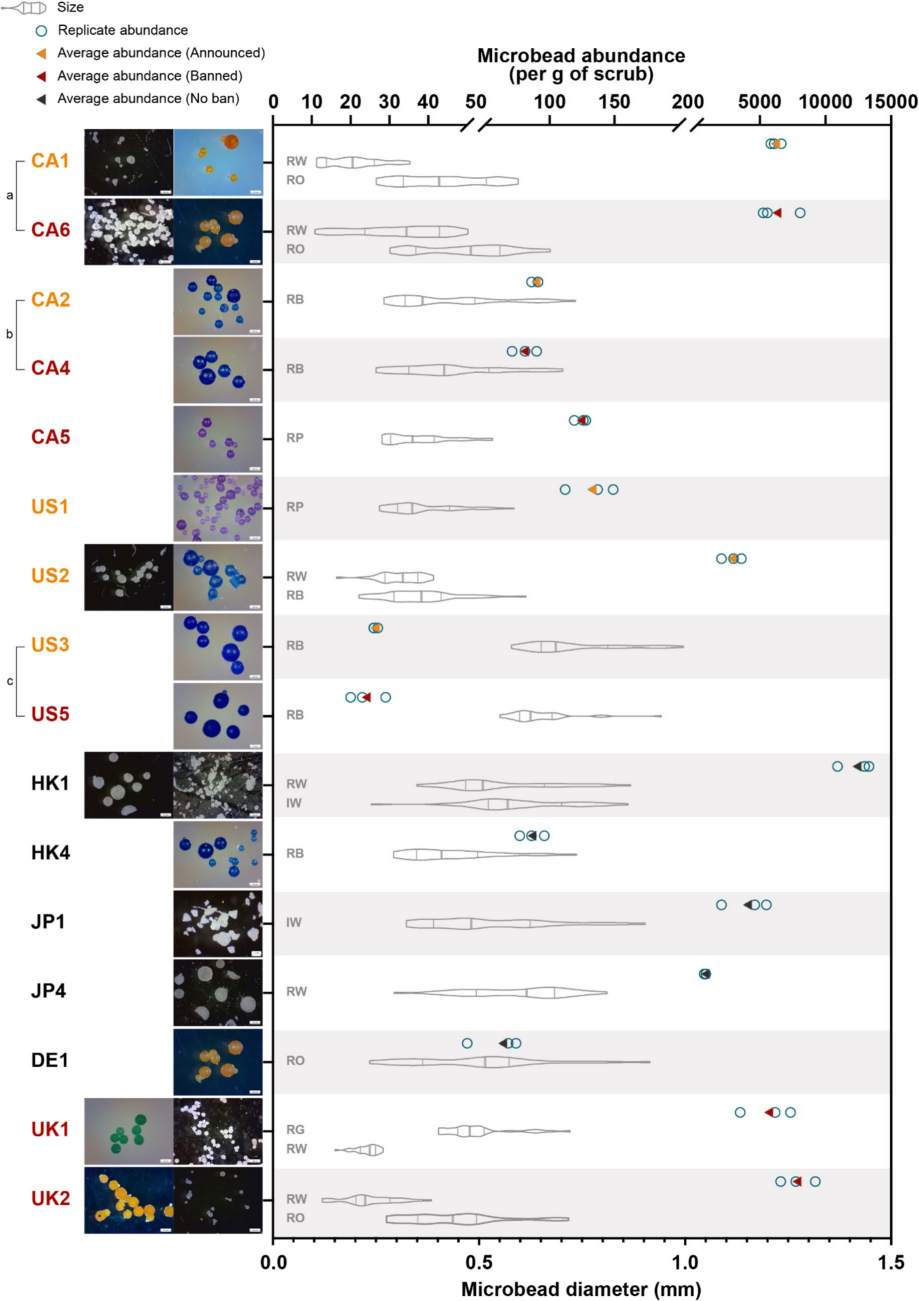
Images of microbeads (left axis), microbead abundance per gram of facial scrub (top axis), and size distribution of microbeads in millimeters (bottom axis) for each facial scrub sample. Facial scrub samples were labelled in black to represent the purchasing regions with no ban, orange for regions with bans announced but yet to be implemented, and red for regions with full ban implementation. a, b, c: Paired products, which were the same products from the same brand purchased in the same region before and after microbead bans were enacted. Types of microbeads were abbreviated as Shape_Colour (e.g. ‘RO’ denotes round orange microbeads). Shapes were abbreviated as ‘R’ for spherical microbeads and ‘I’ for irregularly shaped microbeads. Colours were abbreviated as follows: ‘O’ for orange; ‘W’ for white; ‘B’ for blue; ‘P’ for purple; ‘G’ for green

Thus, of the 28 bottles of facial scrub tested, 12 bottles (43%) did not contain microbeads (HK2, HK3, JP2, JP3, DE2, DE3, CA3, US4, IT1, IT2, UK3, UK4; Table 1), while 16 bottles (57%) were found to contain microbeads. Of these 16 bottles, six bottles contained only a single type of microbeads (CA2, CA4, HK4, JP1, JP4, DE1), six bottles contained a mixture of two types of microbeads (CA1, CA6, US2, HK1, UK1, UK2), and the remaining four bottles contained a mixture of microbeads and non-plastic exfoliants (CA5, US1, US3, US5) (Table 2). In the 16 bottles, the concentration of microbeads ranged from 24 ± 5 (US5) to 12,284 ± 1300 (HK1) beads per gram of facial wash (Table 2), and the size of microbeads ranged from 0.194 ± 0.065 mm up to 0.724 ± 0.114 mm (Table 2; Fig. 1).

## Discussion

### Exfoliant composition

Through composition analysis of our facial scrub samples, we identified natural organic materials including cotton, flax, and cellulose as the exfoliant in 16 out of 28 facial scrubs (Table 2). This finding aligns with the new market trends that biodegradable alternatives are replacing microbeads (Venus 2020). However, these natural alternatives only comprised 48% of the types of facial exfoliants present in this study (20 out of 42 types), while the remaining were identified as microbeads (52%, 22 out of 42 types) (Table 2).

Microbeads identified in this study are all composed of synthetic polymers, and can be classified into two groups: conventional plastics and plastic-like polymers. As suggested by FTIR and Raman analyses, the major types of microbeads (82%, 18 out of 22 types) extracted in this study appear to be composed of conventional plastics that include PE (CA1, CA2, CA4-CA6, US2, US3, US5, HK4, JP1, DE1, UK1, UK2), olefin (JP4), and copolymer EPDM or polyvinyl stearate/PE (UK1). Meanwhile, the remaining (18%, 4 out of 22 types) are composed of plastic-like polymers, including PE wax (CA1), microcrystalline wax (US1), and tetracontane (HK1) (Table 2). Noticeably, microbeads remain dominant in facial scrubs in regions with microbead ban implemented, with more than half of their exfoliants found to be microbeads (64%, 9 out of 14 types) (Table 1; Table 2). Our results demonstrate the prevalent use of microbeads as facial exfoliants despite the existence of natural alternatives, implying the need for stronger legislative or market initiatives to drive the material shift from microbeads to non-plastic exfoliants in these products.

### Microbead abundance in facial scrubs

The quantity of microbeads in facial scrubs varied greatly where almost half of the products tested (43%) did not contain any microbeads. For the remaining products that were found to contain microbeads (57%), we noticed that these products could be roughly categorized into those that contained relatively low quantities of microbeads versus products that contained a much higher abundance (Table 1; Fig. 1). The lower concentration products generally contain around 100 microbeads per gram of facial wash. In contrast, the high-concentration products contain thousands of microbeads per gram. Our results show that more than 96% of the microbeads are present in just seven products (HK1, JP1, CA1, US2, CA6, UK1, UK2), while all of these high concentration products except JP1 contain two types of microbeads (Fig. 1). This finding suggests that a small number of products may play a dominant role in the release of microbeads into the environment and targeting these specific products could have a big impact in efforts to combat microbead pollution.

### Effect of microbead bans

Our study reveals the persistence of microbeads in products across different implementation stages of microbead bans (Table 1). In particular, microbeads are detected in six out of eight products in regions with microbead bans in place, with concentrations ranging from 24 ± 5 to 6298 ± 1543 beads per gram of facial wash, which suggests the limited influence of microbead bans. This finding is supported when we compare the paired products (products from identical brands and regions purchased prior to and following ban implementation) as microbeads were found to be persistently present (Table 1). In addition, the consistent concentration, colour, shape, size distribution, and polymer composition of microbeads in these products (Table 2; Fig. 1) also indicate the limited effectiveness of bans to induce market shifts from plastics to natural alternatives.

Interestingly, some brands produced products that were consistently microbead-free regardless of the state of any legislation (Table 1). Specifically, products from Brand 3 and Brand 5 only utilized natural alternatives such as cotton, flax, and cellulose as exfoliants in their products. It is worth noting that these brands have made public commitments to environmental, social, and governance (ESG) issues, indicating that market incentives may play an important role in driving changes towards more sustainable product production (Dauvergne 2018; Diana et al. 2022).

### Broadening and unifying ban coverage

The two groups of microbeads identified in this study—conventional plastics and plastic-like polymers—vary in their market restrictions despite their similar chemical compositions. Microbeads produced with conventional plastics including PE, PP, and PET are widely banned in countries with legislation in place. Meanwhile, the banning of plastic-like polymers (for example, synthetic waxes) is still under debate as these materials are more likely to melt at lower temperature and are utilized as ‘liquid polymers’ by manufacturers (Munno et al. 2018; Dauvergne 2018).

In fact, synthetic wax is a long petroleum-based polymer that closely resembles PE. Despite the wide adoption of FTIR to identify plastics, distinguishing between wax and PE by FTIR can be challenging due to their similar compositions (Habib et al. 2020). This is also demonstrated in the present study as all conventional plastic microbeads we detected (mainly PE) were listed as plastic-like polymers on product ingredient lists, namely ‘synthetic wax’ (in CA1, CA6, US2, US3, US5, UK2), ‘microcrystalline wax’ (in CA5, US2, HK4), ‘cera microcristallina’ (in CA1, CA2, CA4, CA6, DE1, UK1, UK2), and ‘paraffin’ (in JP1, JP4) (Table S1). The clear mismatch between the labelled and the identified microbead compositions suggests that PE and synthetic wax have very similar compositions that can hardly be differentiated. On the other hand, a previous study by Akhbarizadeh et al. (2024) suggested that PE microbeads are primarily irregularly shaped while PE wax are primarily spherically shaped. However, our study shows that a majority of round-shaped microbeads are composed from PE (or olefin) (Table 2), which does not align with previous findings and indicates that distinguishing wax and PE in facial products by their shapes may not be practical. Given that one group of microbeads (conventional polymers) is banned but the other group (plastic-like polymers) is not, the difficulty in differentiating the two by their chemical compositions and physical properties may pose challenges in monitoring manufacturers’ compliance to microbead bans.

Synthetic wax (namely PE wax, microcrystalline wax, cera microcristallina, and paraffin) is commonly used as an exfoliant in personal care products. For instance, the present study and other studies have detected wax in facial wash products (Cheung and Fok 2016; Nawalage and Bellanthudawa 2022; Jasmine et al. 2024; Akhbarizadeh et al. 2024), indicating its prevalence as an exfoliant. Yet, given its extremely similar chemical composition and physical properties to conventional plastic microbeads in facial scrubs, synthetic wax is surprisingly not listed in any of the bans.

According to Dauvergne (2018), the PCCP industry views wax as a semi-liquid ingredient applied in cosmetics, which is not considered a plastic as long as it is not in a solid form, as defined by U.S. federal law (Strifling 2016). However, given the fact that synthetic wax has a lower melting point than conventional plastics, their potential impacts as a plastic pollutant cannot be ignored. According to the United Nations Food and Agriculture Organization (1995), the melting point of microcrystalline wax ranges from 62 to 102 °C, which closely resembles the findings of Habib et al. (2020) that softening point of microbeads in cosmetic products ranges from 65 to 123 °C. This implies that synthetic wax microbeads may remain as solids (i.e. plastics) in real-life scenarios. In particular, they may survive wastewater treatment plant (WWTP) treatment, which typically operates at temperatures ranging from 20 to 35 °C, and be subsequently discharged into the marine environment. Therefore, there is a crucial need to develop a better understanding of the potential ecological impacts of synthetic wax as well as to consider including synthetic wax in ban legislation.

Indeed, the current scope of ban legislation varies significantly between regions, leading to a lack of clear guidelines for international manufacturers on which materials to avoid. For example, South Korea has banned the 22 plastic components listed by UNEP (2015) in facial scrubs, while the US and France have banned bioplastic alternatives (Venus 2020). Hence, it is crucial to refine the scope of microbead bans to facilitate mutual understanding and ensure collective efforts towards combating the targeted pollutants. Providing more specific descriptions on microbead compositions and physical characteristics will facilitate the manufacturers’ comprehension and enhance their capacity to comply with microbead bans and formulate microbead-free products.

## Conclusion

Our study reveals that microbeads are still present in facial products after microbead ban implementation, indicating the need for further actions. We have identified a small number of products that contribute significantly to microbead release, suggesting that targeting these specific products could have a significant impact on reducing microbead pollution. To improve microbead ban effectiveness, our study suggests expanding the scope of microbead bans to include other plastic-like polymers, such as synthetic wax (commercially labelled as ‘synthetic wax’, ‘microcrystalline wax’, ‘cera microcristallina’, and ‘paraffin’), which are commonly used as an alternative to conventional plastic microbeads. These plastic-like microbeads not only share a similar chemical composition to conventional microbeads but may also be discharged as solid particles much like conventional microbeads. As their environmental impacts are yet to be determined, a precautionary approach to the management of these particles would be recommended. Additionally, the use of natural exfoliants as a replacement to both these plastic-like and conventional microbeads should be prioritized to ensure no further harm to the environment. Meanwhile, it is essential to establish global consensus on microbeads to provide clear guidelines for international manufacturers and to achieve the shared goal of eliminating microbeads from personal care and cosmetic products globally.

## Supplementary Information

Below is the link to the electronic supplementary material.

Supplementary file1 (DOCX 1091 KB)

Supplementary file2 (PDF 224 KB)

## Data Availability

The authors declare that the data supporting the findings of this study are available within the paper and its supplementary information files. Datasets generated during the current study are available from the corresponding author on reasonable request.
