# Supplementary material for: State of microbeads in facial scrubs: persistence and the need for broader regulation
Source: Environ Sci Pollut Res Int. 2025 Apr 7;32(17):11063–71. doi: 10.1007/s11356-025-36341-3 (PMC12014694; doi:10.1007/s11356-025-36341-3)
Supplement: Supplementary file 1 — Supplementary file1 (DOCX 1091 KB) [file 11356_2025_36341_MOESM1_ESM.docx]

**Supplementary material:**

**Chemical composition of microbeads identified by FTIR and verified by Raman spectroscopy**

**CA1 RW**

FTIR

**
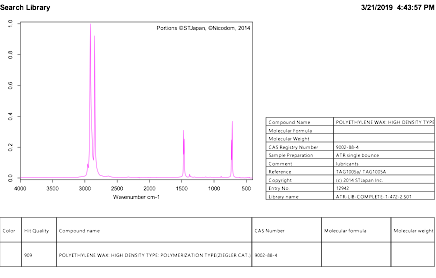
**

Raman

**
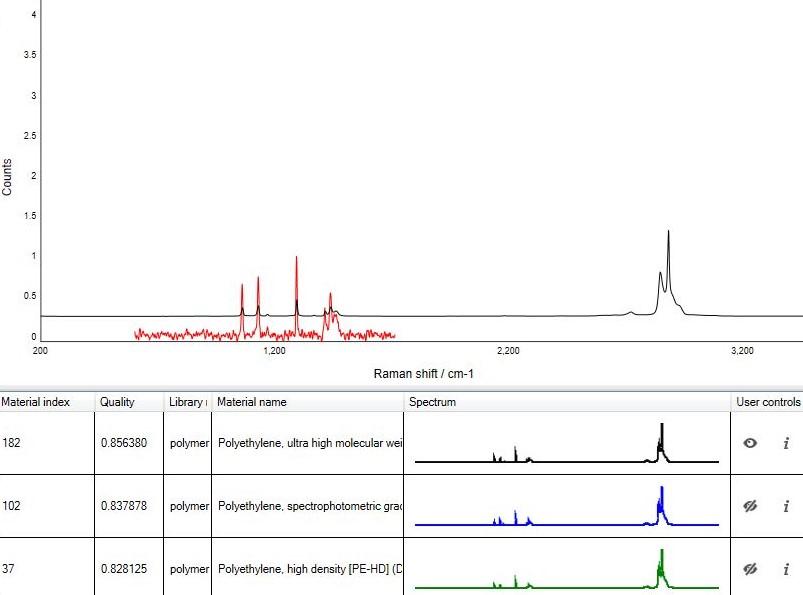
**

**CA1 RO**

FTIR

**
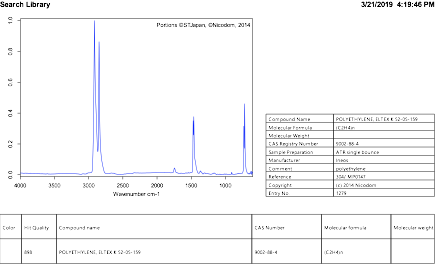
**

Raman

**
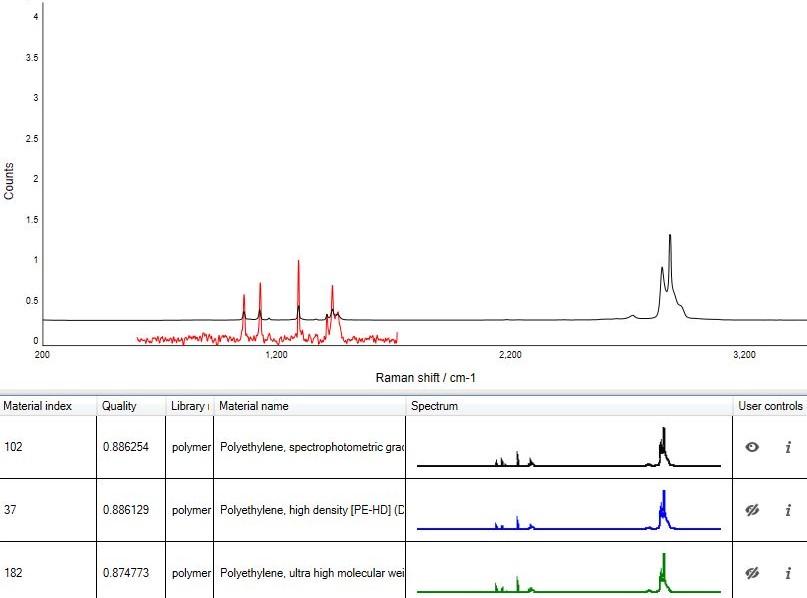
**

**CA2 RB**

FTIR

**
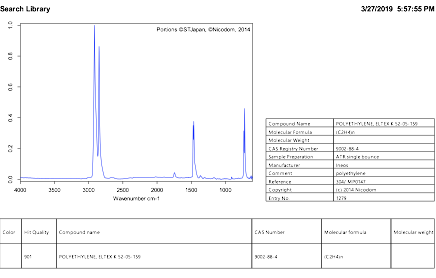
**

Raman

**
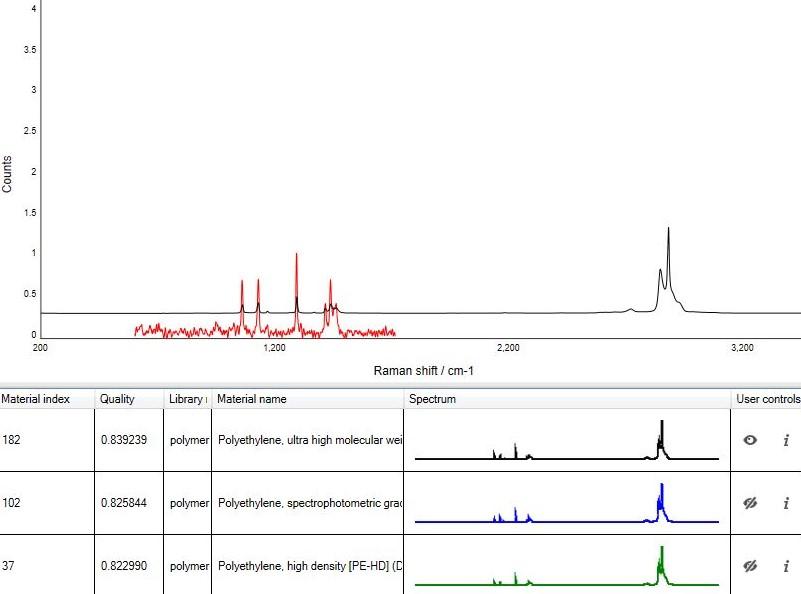
**

**CA4 RB**

FTIR

**
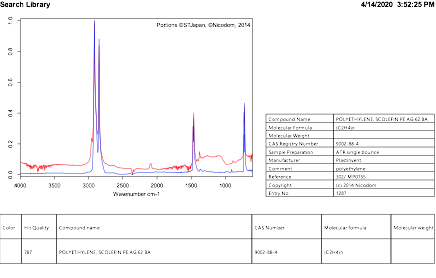
**

Raman

**
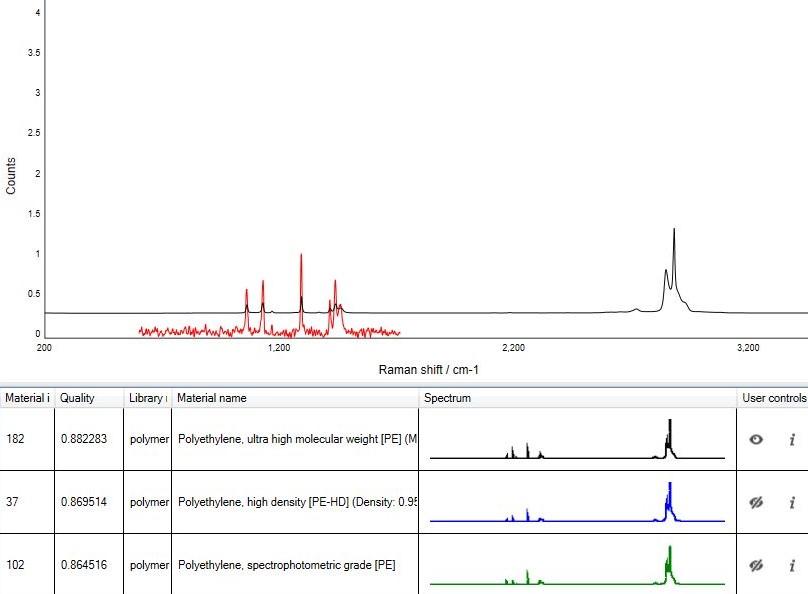
**

**CA5 RP**

FTIR

**
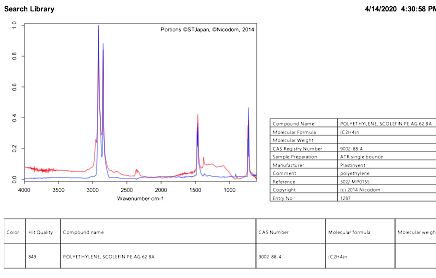
**

Raman

**
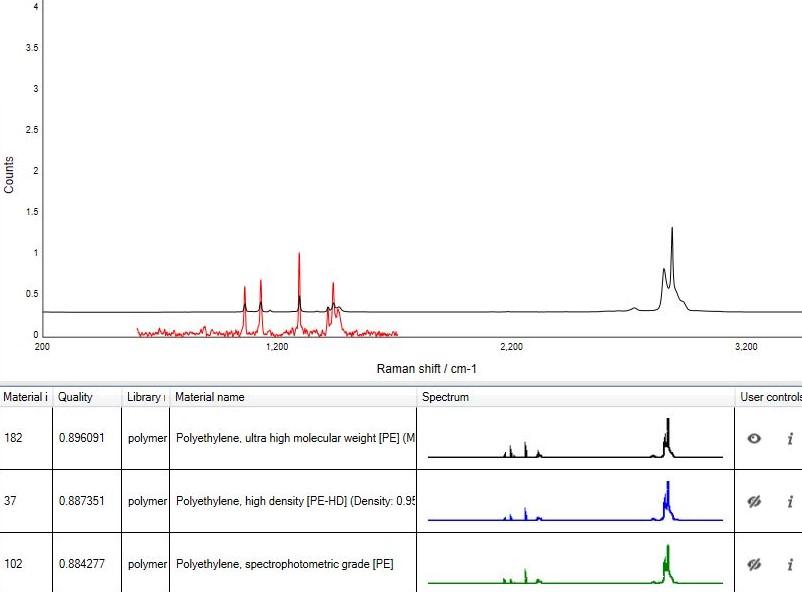
**

**CA6 RO**

FTIR

**
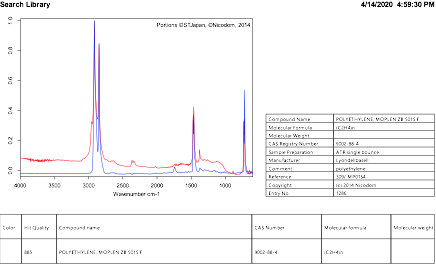
**

Raman

**
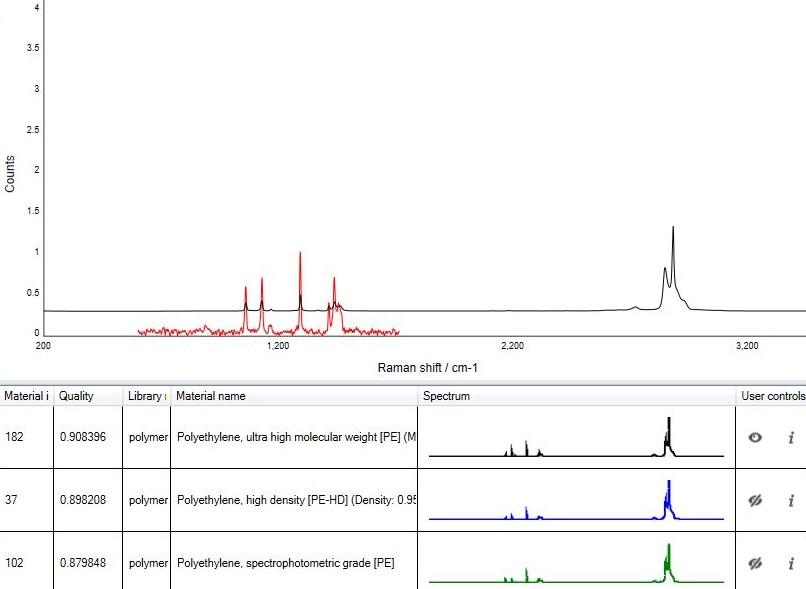
**

**CA6 RW**

FTIR

**
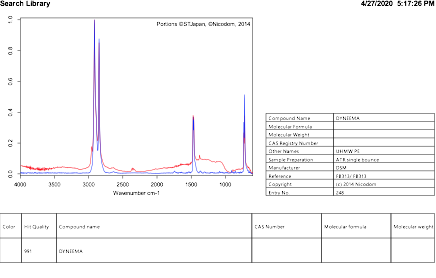
**

Raman

**
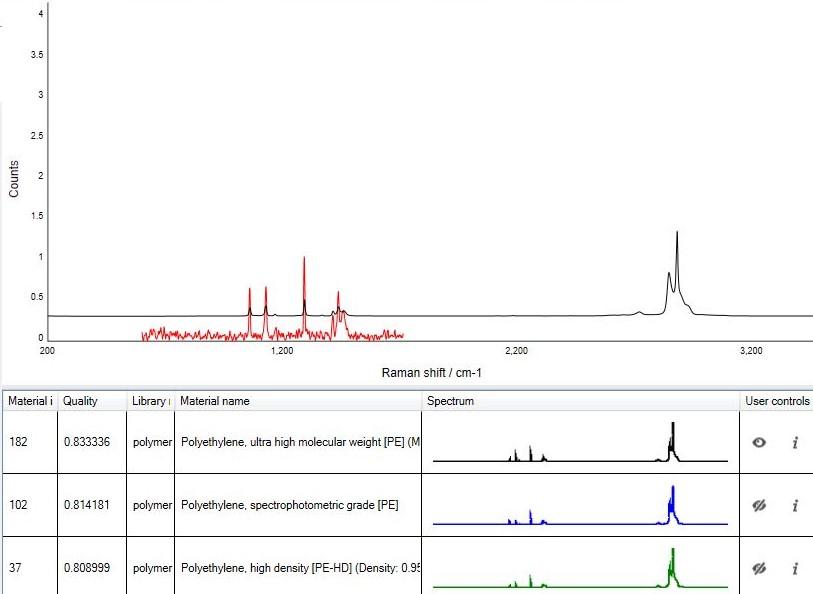
**

**US1 RP**

FTIR

**
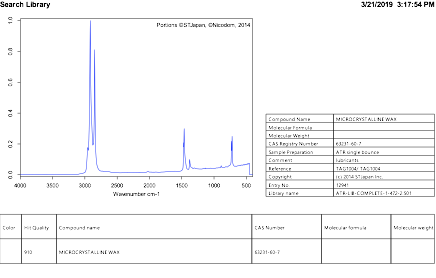
**

Raman

**
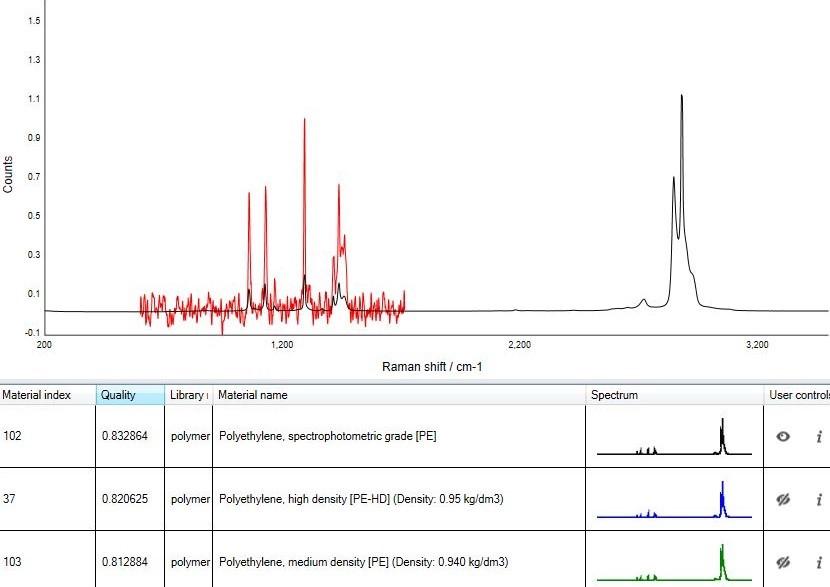
**

**US2 RW**

FTIR

**
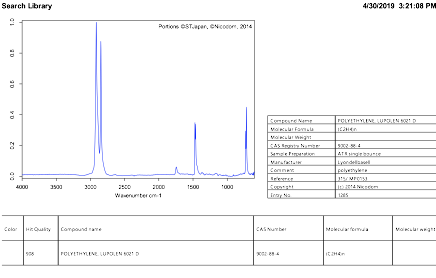
**

Raman

**
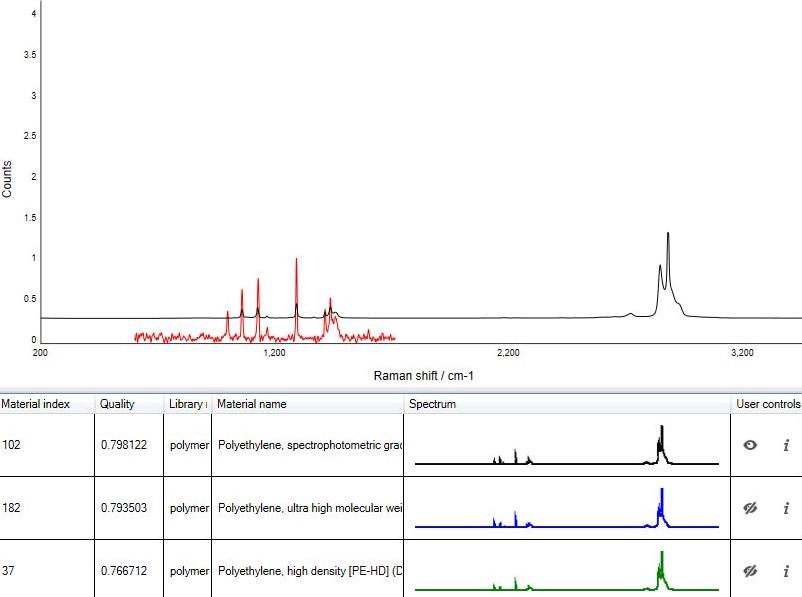
**

**US2 RB**

FTIR

**
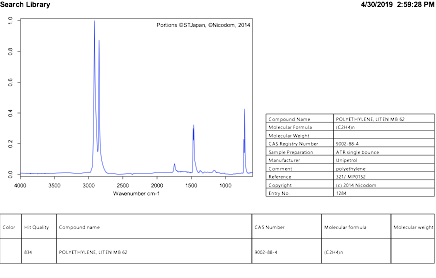
**

Raman

**
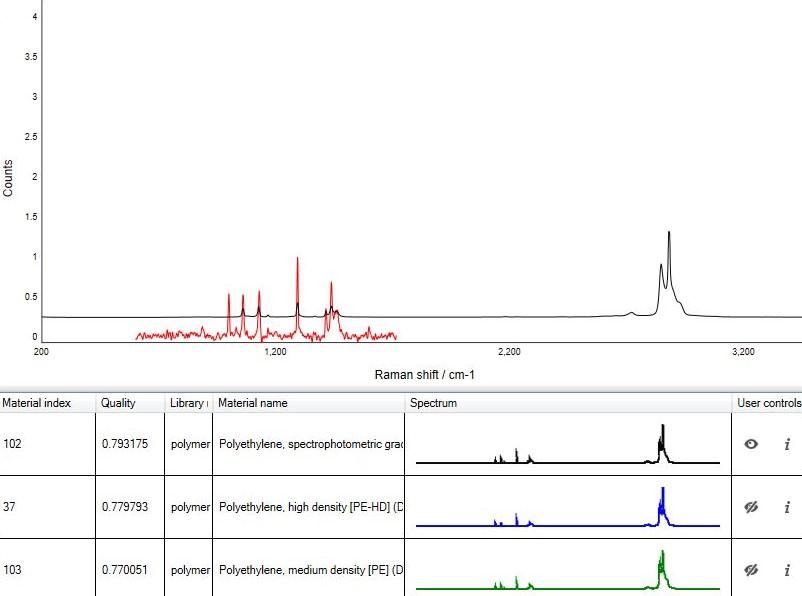
**

**US3 BR**

FTIR

**
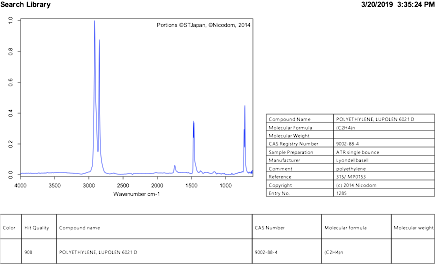
**

Raman

**
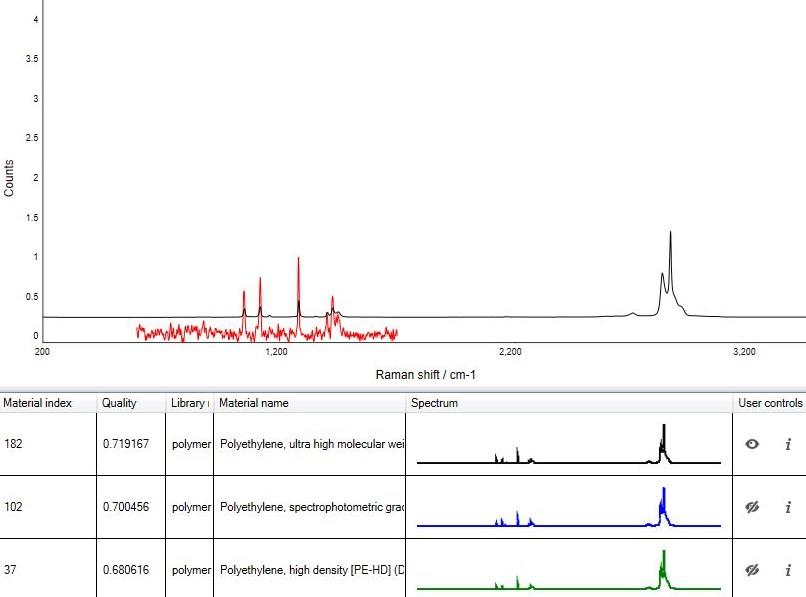
**

**US5 RB**

FTIR

**
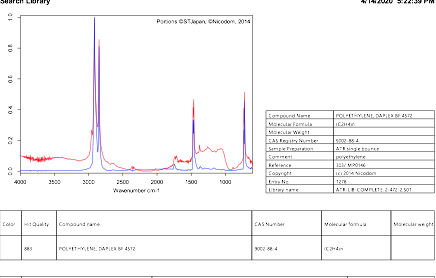
**

Raman

**
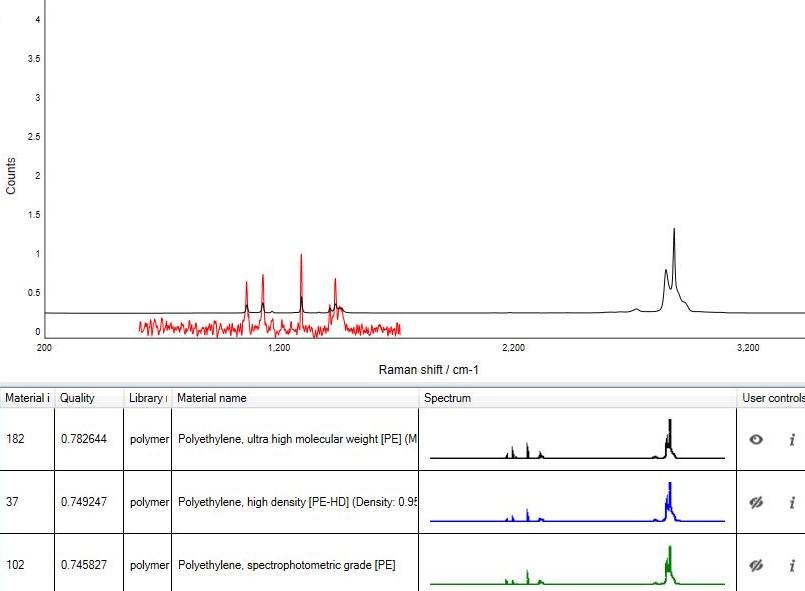
**

**HK1 RW**

FTIR

**
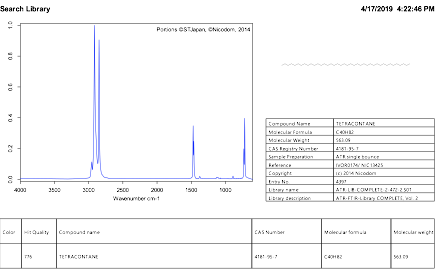
**

Raman

**
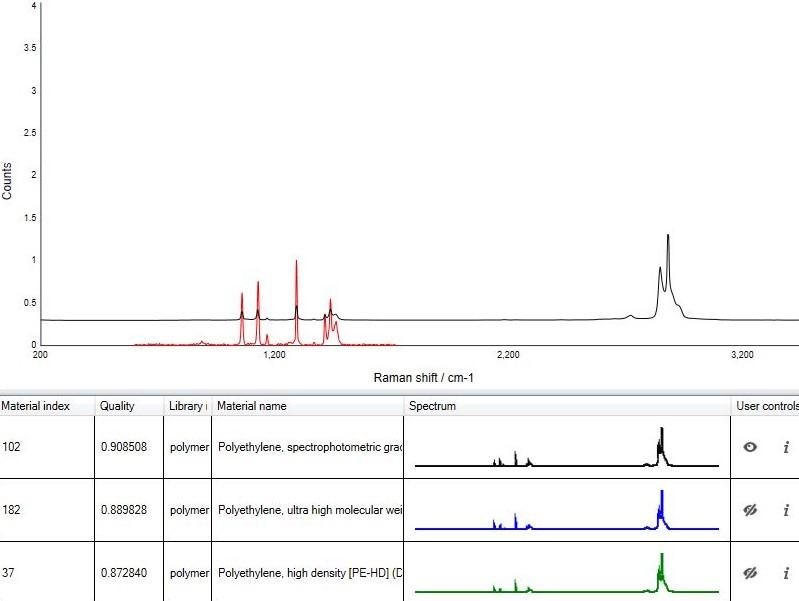
**

**HK1 IW**

FTIR

**
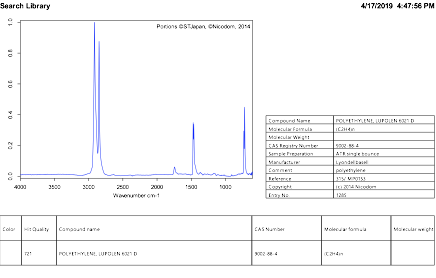
**

Raman

**
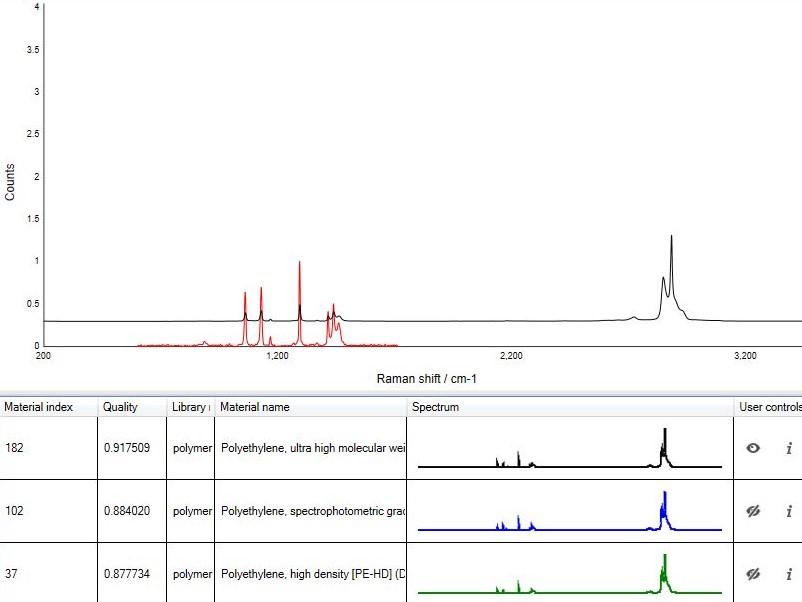
**

**HK4 RB**

FTIR

**
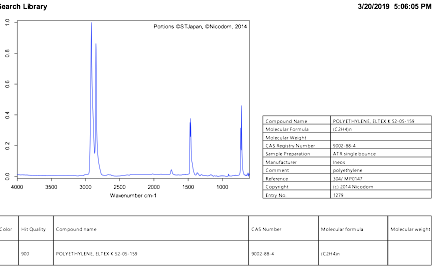
**

Raman

**
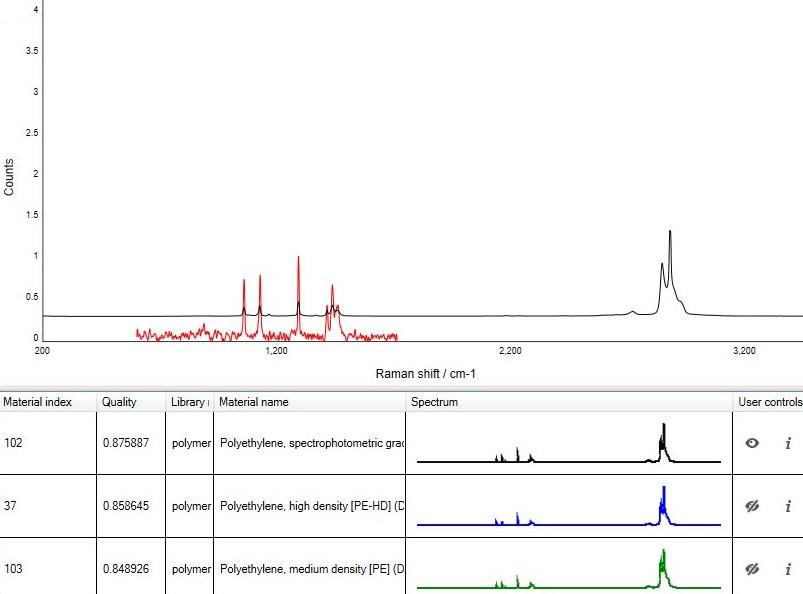
**

**JP1 WI**

FTIR

**
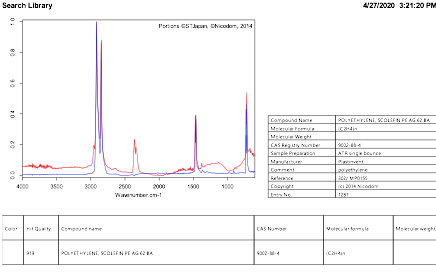
**

**JP4 RW**

FTIR

**
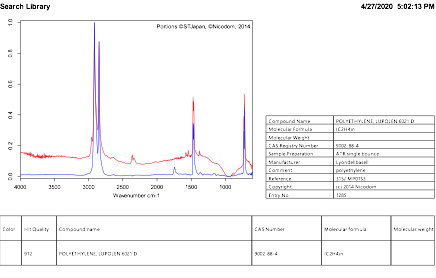
**

Raman

**
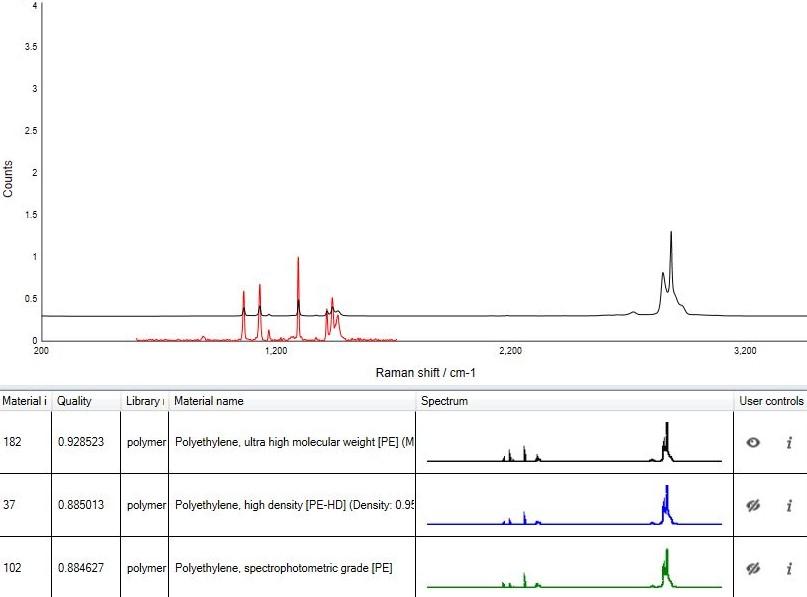
**

**DE1 RO**

FTIR

**
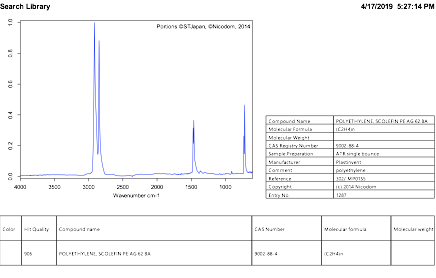
**

Raman

**
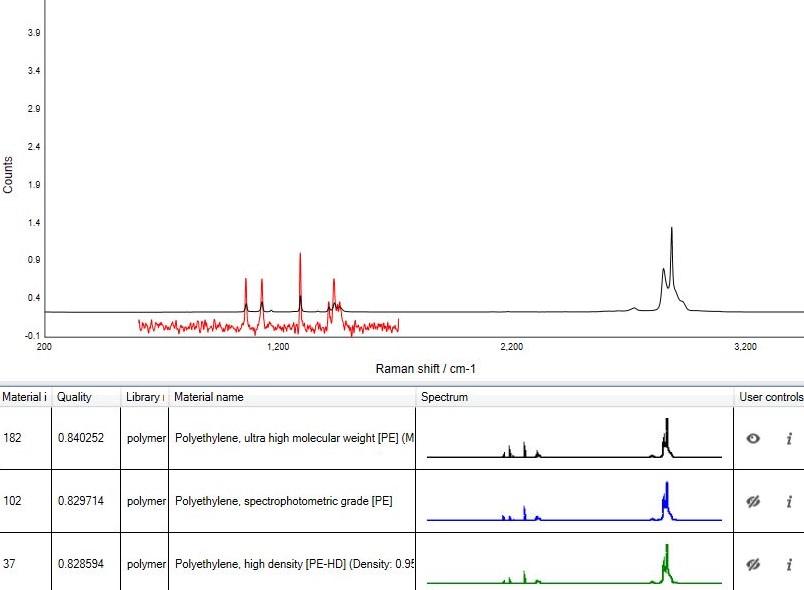
**

**UK1 RG**

FTIR

**
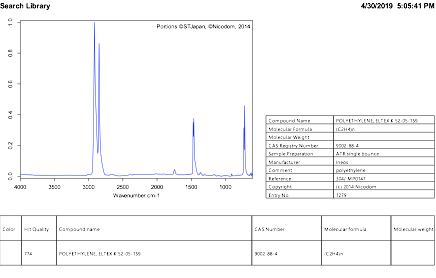
**

Raman

**
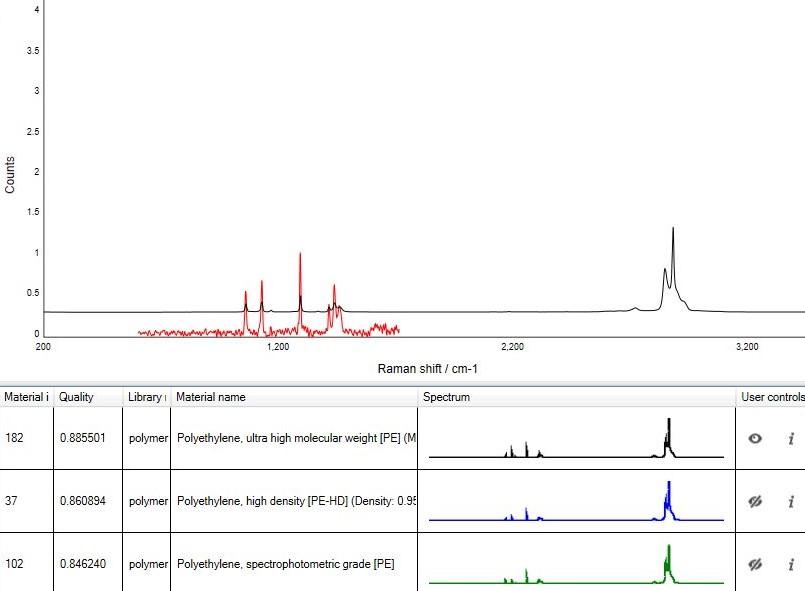
**

**UK1 RW**

FTIR

**
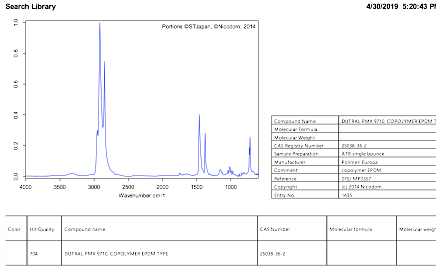
**

Raman

**
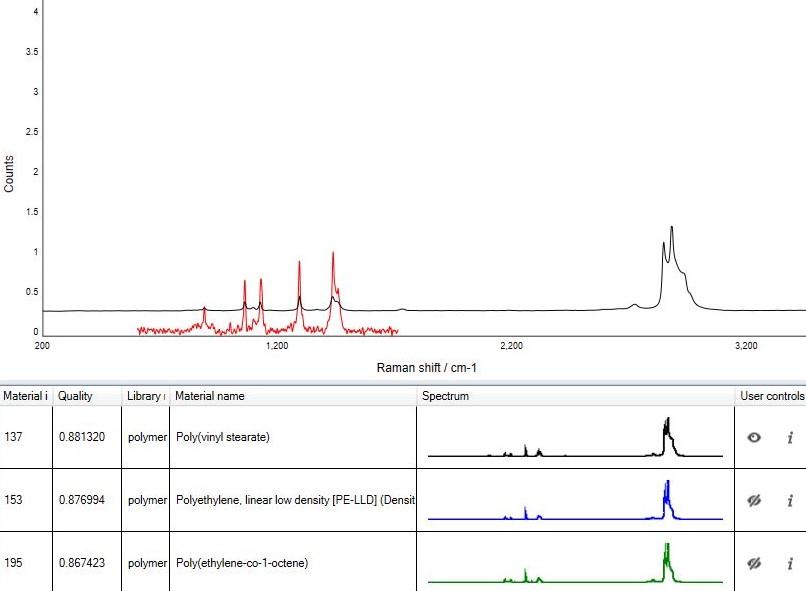
**

**UK2 RW**

FTIR

**
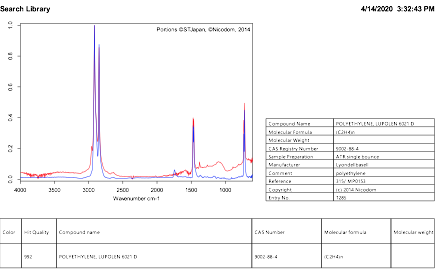
**

Raman

**
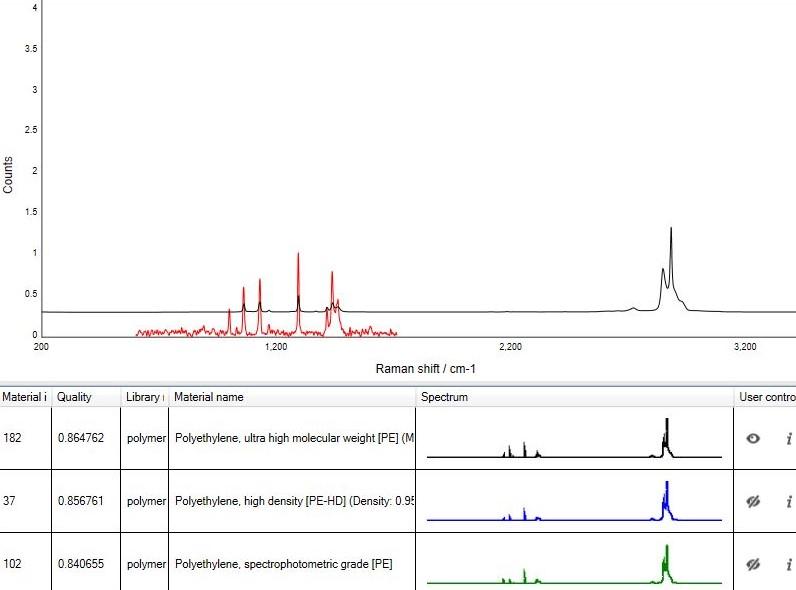
**

**UK2 RO**

FTIR

**
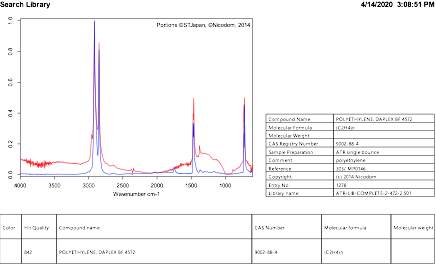
**

Raman

**
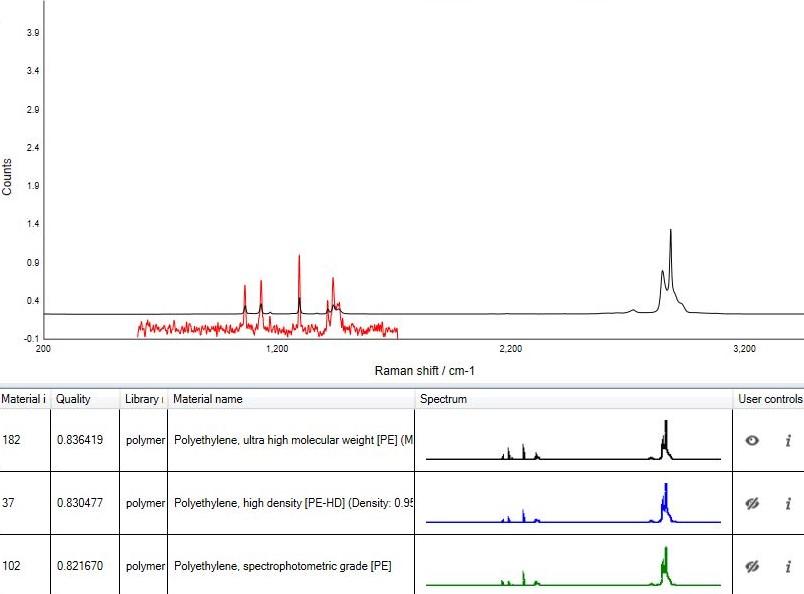
**
