# Supplementary material for: State of microbeads in facial scrubs: persistence and the need for broader regulation
Source: Environ Sci Pollut Res Int. 2025 Apr 7;32(17):11063–71. doi: 10.1007/s11356-025-36341-3 (PMC12014694; doi:10.1007/s11356-025-36341-3)
Supplement: Supplementary file 2 — Supplementary file2 (PDF 224 KB) [file 11356_2025_36341_MOESM2_ESM.pdf]

Table S1. Comparison between the composition of exfoliants extracted from the 28 samples and the exfoliant composition listed on the product labels. Facial scrub samples were labelled in black to present the purchasing regions with no ban, orange for regions with bans announced but yet to be implemented, and red for regions with full ban implementation.

Sample	Total no. of types of exfoliants	No. of types of microbeads	Exfoliants characteristics						Skeptical plastic exfoliants identified on product label	Natural exfoliants identified on product label
			Microbead	Colour	Shape	Polymer identification by FTIR	Polymer verification by Raman	Average microbead size (mm)		
CA1[a]	2	2	✓	White	Round	Polyethylene wax	PE	0.194 ± 0.065	Synthetic wax, Cera microcristallina	Charcoal powder
			✓	Orange	Round	PE	PE	0.413 ± 0.107		
CA2[b]	1	1	✓	Blue	Round	PE	PE	0.427 ± 0.133	Cera microcristallina	Cellulose
CA3	2	0		Blue	Round	Cotton + flax (60:40)	-	-	-	Microcrystalline cellulose, Cellulose, Lactose
				White	Round	Cotton	-	-		
CA4[b]	1	1	✓	Blue	Round	PE	PE	0.432 ± 0.124	Cera microcristallina	Cellulose
CA5	2	1	✓	Purple	Round	PE	PE	0.352 ± 0.077	Microcrystalline wax	Microcrystalline cellulose, Cellulose
				White	Round	Cotton flax	-	-		
CA6[a]	2	2	✓	Orange	Round	PE	PE	0.466 ± 0.111	Synthetic wax, Cera microcristallina	Charcoal powder
			✓	White	Round	Dyneema/ PE	PE	0.305 ± 0.109		
US1	2	1	✓	Purple	Round	Microcrystalline wax	PE	0.364 ± 0.085	Microcrystalline wax	Microcrystalline cellulose, Cellulose
				White	Round	Cellulose swab	-	-		
US2	2	2	✓	White	Round	PE	PE	0.309 ± 0.053	Synthetic wax, Microcrystalline wax	-
			✓	Blue	Round	PE	PE	0.361 ± 0.092		
US3[c]	2	1	✓	Blue	Round	PE	PE	0.724 ± 0.114	Synthetic wax	Microcrystalline cellulose, Cellulose
				White	Round	Cellulose swab	-	-		
US4	1	0		Blue	Round	Castor oil, hydrogenated	-	-	-	Hydrogenated castor oil, Charcoal powder
US5[c]	2	1	✓	Blue	Round	PE	PE	0.648 ± 0.08	Synthetic wax	Microcrystalline cellulose, Cellulose
				White	Round	Cellulose swab	-	-		
HK1	2	2	✓	White	Round	Tetracontane	PE	0.555 ± 0.131	Paraffin	-
			✓	White	Irregular	Tetracontane	PE	0.593 ± 0.130		
HK2	1	0		Orange	Irregular	Agarose	-	-	-	Cellulose, Agar, Mica
HK3	2	0		Black	Irregular	Cotton + flax (60:40)	-	-	N/A (list unavailable)	N/A (list unavailable)
				White	Irregular	Cotton	-	-		
HK4	1	1	✓	Blue	Round	PE	PE	0.436 ± 0.113	Micrystalline wax	Cellulose
JP1	1	1	✓	White	Irregular	PE	PE	0.525 ± 0.152	Paraffin	-
JP2	1	0		Orange	Round	PFR Rayon, Rayon fiber	-	-	-	Cornstarch, Cellulose
JP3	1	0		White	Round	1,2,3-propantyl tridocosanoate	-	-	-	Hydrogenated castor oil, Cellulose, Sea silt
JP4	1	1	✓	White	Round	Vexar plastic netting, Olefin fiber	PE	0.590 ± 0.125	Paraffin	Charcoal
IT1	2	0		Blue	Round	Cotton + flax (60:40)	-	-	-	Hydrogenated castor oil, Microcrystalline cellulose
				White	Round	Cotton + flax (60:40)	-	-		
IT2	1	0		Blue	Round	Castor oil, hydrogenated	-	-	-	Hydrogenated castor oil, Corn starch, Pumice
DE1	1	1	✓	Orange	Round	PE	PE	0.499 ± 0.16	Cera microcristallina	-
DE2	1	0		Blue	Round	Mobil SHC (synthetic for high temperature, roiling oil)	-	-	-	Hydrogenated castor oil, Silica
DE3	1	0		Blue	Round	Cotton	-	-	-	Hydrogenated castor oil, Microcrystalline cellulose, Cellulose
UK1	2	2	✓	Green	Round	PE	PE	0.500 ± 0.083	Cera microcristallina	Hydrogenated castor oil
			✓	White	Round	Copolymer EPDM	Polyvinyl stearate/ PE	0.229 ± 0.027		
UK2	2	2	✓	White	Round	PE	PE	0.237 ± 0.065	Cera microcristallina, Synthetic wax	Charcoal powder
			✓	Orange	Round	PE	PE	0.438 ± 0.118		
UK3	1	0		Blue	Round	Pantone black natural 10009 (dried)	-	-	-	Hydrogenated castor oil
UK4	2	0		Blue	Round	Cotton + flax (60:40)	-	-	-	Microcrystalline Cellulose, Lactose, Cellulose
				White	Round	Cellulose swab	-	-		
